# The Institutional Library

**Published:** 1921-07-30

**Authors:** 


### THE INSTITUTIONAL LIBRARY.

**Medical Conduct and Practice.** By W. G. Aitchison Robertson, M.D., D.Sc. (A. and C. Black, Ltd., London. Price 6s. net.)

THE subject of medical ethics has claimed no little attention of late from both the General Medical Council and the Ethical Committee of the British Medical Association. It is a most desirable reform that students preparing for their final examinations should be actually instructed in this subject instead of trusting to chance and the acuteness of their wits. One is glad, therefore, to see an excellent little book in which Dr. Aitchison Robertson, of Edinburgh, has embodied much of the sound advice his students always receive. Many of us will feel, as we read his entertaining chapters, that we were indeed unfortunate not to have had these truths explained before we stumbled unguided among the pitfalls of early practice. At least we can advise those who come after us to profit by the help this little book can give.

**Diseases of the Nervous System.** By H. CAMPBELL THOMSON, M.D., F.R.C.P. Third edition. (Cassell & Co. 1921. Pp. 566. Price 15s. net.)

SINCE the second edition of this text-book was published the war has resulted in considerable modifications of many neurological concepts and doctrines. With these Dr. Thomson has duly reckoned; he has incorporated those of them that are likely to bear the test of time in his monograph. We retain the exceedingly favourable impression made on former occasions by this book, and know of no better volume of reference for the general practitioner in the specialty with which it deals. The price, too, has not soared so alarmingly as in the case of several other text-books; and the format seems to be just as pleasing as that of pre-war days.

**Lippincott's Quick Reference Book for Medicine and Surgery.** By GEORGE E. REHBERGER, A.B., M.D. (J. B. Lippincott Co. Price 63s. net.)

THIS ample volume really comprises eleven books in one, for besides dealing with general medicine and surgery it is a dictionary of gynæcology, genito-urinary diseases, obstetrics, skin diseases, as well as diseases of the eye, ear, nose, and throat, orthopædics and drugs, compiled and presented in such a manner as to appeal most to the general practitioner in need of a handy and reliable book of reference, though a student on the eve of an examination would also find it most useful for refreshing his memory on important points. Each disease is treated alphabetically under its appropriate heading, and in a complete, though necessarily brief, manner is then given the definition of the disease, next the enumeration of all its possible causes, then its prognosis, probable duration, outcome, and treatment. Every separate part ends with an appendix containing a scheme for the history and examination of the patient, and includes a list of all instruments, drugs, and everything required for the proper treatment of each disorder, concisely catalogued for ready reference in emergency. The reader will find himself in hearty agreement with the author when he expresses the opinion that the book "will fill a great need in bedside therapeutics," though modestly claiming nothing as his own in the volume beyond the system, selective judgment employed, and the great amount of work it has entailed. He has certainly undertaken, and with marked success, a veritable labour of Hercules, and has produced a most useful and extremely interesting book. For years himself an "isolated country doctor," he knows thoroughly the needs of those similarly placed, and has endeavoured to cater specially for them.

The excellent plates, diagrams, and illustrations throughout serve to elucidate the text wherever necessary. Methods described of treatment are thoroughly up to date and clearly explained. The names of authorities for treatment prescribed are always given, so that they can, if desired, be consulted in full. The printing and paper are both good, though the binding is scarcely strong enough for a volume of such great bulk. In a future edition this defect will no doubt be remedied.

**Barrier Chart for Health Officers.** By S. H. DAUKES, O.B.E., M.D., M.B. (Bailliere, Tindal & Cox. Price 3s. 6d. net.)

OWING to the growth in our population, especially in towns, it is increasingly important in the prevention of that ever-widening group of diseases known broadly as "communicable" that no known method of prevention be neglected. In communicable diseases two points must be grasped, viz.. the mode of entrance of the virus, and the path of its exit from the body, and keeping these two points in view it is not difficult to classify most diseases. The publication now before us aims at such a classification. It is presented in the form of four charts covering all the ordinary communicable diseases. Chart No. I. is the alimentary group, and includes diseases such as enteric and parasitic diseases which find an entrance by the mouth; Chart II. is the inoculation group, mostly by bites of parasites; Chart III. the respiratory group, such as measles; and Chart IV. the contact group, such as syphilis and anthrax. Each chart is again arranged in tabular form, and displays succinctly all our present knowledge of each disease, viz., where the infective agent is found, the incubation period, etc. In all this, however, there is nothing new or original, but what the author can claim as new and original is the setting out of the four columns showing what he calls the "barriers," or lines of defence of each disease, with four additional columns giving the results of defective barriers. The first "barrier" deals with the sick person and carriers, the second with intermediate hosts, the third with general hygiene, and the fourth with special immunisation by vaccines, etc. Taken on the whole, the chart should be found most helpful, especially to health officers, who have not always the time or facilities to look up the latest authorities on the prevention of disease.

**Practical Surgical Nursing for Probationers.** By MISS C. SEYMOUR YAPP. Second Impression. (Poor Law Publications, Ltd. Price 3s. 6d.; post 4s.)

THIS is a guide both to the matter and the spirit of nursing. The book is arranged as a series of thirty-six lectures, and the clear table of contents enables a nurse to refer easily to any special point. It is also a convenient handbook for teachers.

